# Population-based trends and risk factors of early- and late-onset preeclampsia in Taiwan 2001–2014

**DOI:** 10.1186/s12884-018-1845-7

**Published:** 2018-05-31

**Authors:** Shu-Han You, Po-Jen Cheng, Ting-Ting Chung, Chang-Fu Kuo, Hsien-Ming Wu, Pao-Hsien Chu

**Affiliations:** 1 Department of Obstetrics and Gynecology, Chang Gung Memorial Hospital, Linkou, Taiwan; 2 Big data research office, Chang Gung Memorial Hospital, Linkou, Taiwan; 3 Division of Rheumatology, Allergy and Immunology, Chang Gung Memorial Hospital, Linkou, Taiwan; 4 Department of Cardiology, Chang Gung Memorial Hospital, Linkou, Taiwan

**Keywords:** Preeclampsia, Incidence, Risk factors, Early onset, Late onset, Hypertension

## Abstract

**Background:**

Preeclampsia, a multisystem disorder in pregnancies complicates with maternal and fetal morbidity. Early- and late-onset preeclampsia, defined as preeclampsia developed before and after 34 weeks of gestation, respectively. The early-onset disease was less prevalent but associated with poorer outcomes. Moreover, the risk factors between early -and late- onset preeclampsia could be differed owing to the varied pathophysiology. In the study, we evaluated the incidences, trends, and risk factors of early- and late- onset preeclampsia in Taiwan.

**Methods:**

This retrospective population-based cohort study included all ≧20 weeks singleton pregnancies resulting in live-born babies or stillbirths in Taiwan between January 1, 2001 and December 31, 2014 (*n* = 2,884,347). The data was collected electronically in Taiwanese Birth Register and National Health Insurance Research Database. The incidences and trends of early- and late-onset preeclampsia were assessed through Joinpoint analysis. Multivariate logistic regression was used to analyze the risk factors of both diseases.

**Results:**

The age-adjusted overall preeclampsia rate was slightly increased from 1.1%(95%confidence interval [CI], 1.1–1.2) in 2001 to 1.3% (95%CI, 1.2–1.3) in 2012 with average annual percentage change (AAPC) 0.1%/year (95%CI, 0–0.2%). However, the incidence was remarkably increased from 1.3% (95%CI, 1.3–1.4) in 2012 to 1.7% (95%CI, 1.6–1.8) in 2014 with AAPC 1.3%/year (95%CI,0.3–2.5). Over the study period, the incidence trend in late-onset preeclampsia was steadily increasing from 0.7% (95%CI, 0.6–0.7) in 2001 to 0.9% (95%CI, 0.8–0.9) in 2014 with AAPC 0.2%/year (95%CI, 0.2–0.3) but in early-onset preeclampsia was predominantly increase from 0.5% (95%CI, 0.4–0.5) in 2012 to 0.8% (95%CI, 0.8–0.9) in 2014 with AAPC 2.3%/year (95%CI, 0.8–4.0). Advanced maternal age, primiparity, stroke, diabetes mellitus, chronic hypertension, and hyperthyroidism were risk factors of preeclampsia. Comparing early- and late-onset diseases, chronic hypertension (ratio of relative risk [RRR], 1.71; 95%CI, 1.55–1.88) and older age (RRR, 1.41; 95%CI 1.29–1.54) were more strongly associated with early-onset disease, whereas primiparity (RRR 0.71, 95%CI, 0.68–0.75) had stronger association with late-onset preeclampsia.

**Conclusions:**

The incidences of overall, and early- and late-onset preeclampsia were increasing in Taiwan from 2001 to 2014, predominantly for early-onset disease. Pregnant women with older age and chronic hypertension had significantly higher risk of early-onset preeclampsia.

**Electronic supplementary material:**

The online version of this article (10.1186/s12884-018-1845-7) contains supplementary material, which is available to authorized users.

## Background

Worldwide 2.0 to 8.0% pregnancies were complicated by preeclampsia [1–5] with variation across regions [2]. Preeclampsia, the progressive disorder during pregnancy is strongly associated with maternal and fetal complications including eclampsia, acute renal failure, coagulopathy, placenta abruption, postpartum hemorrhage, intrauterine growth restriction, medically indicated preterm birth, and maternal and fetal death [1, 6, 7]. In a systemic analysis from World Health Organization (WHO), hypertensive disorders including preeclampsia accounted for 14.0% maternal death between 2003 and 2009 [8]. Moreover, the risk of severe obstetric morbidities in women with eclampsia or severe preeclampsia was increasing [9]. Although most of the maternal dysfunctions resolved gradually in postpartum, these women were at higher risk of developing chronic hypertension, recurrent preeclampsia in the next pregnancy, and later-life cardiovascular diseases [10].

Preeclampsia is recognized as a heterogenous syndrome with different pathophysiology and be divided in two subtypes according to the disease onset [7, 11, 12]. Early-onset preeclampsia, diagnosed less than 34 gestational weeks was less prevalent than late-onset preeclampsia, occurring at 34 or more weeks of gestation [13]. The incidences of early- and late-onset preeclampsia were 0.3 and 2.7%, respectively [14, 15]; nevertheless, the early-onset disease contributed to more unfavorable maternal and fetal outcomes [14, 16, 17]. Around ten-fold increased risk of perinatal death and maternal death in women with early-onset preeclampsia and twofold higher risk of perinatal death and threefold increased risks of maternal death in women with late-onset disease were observed, comparing with normal pregnancy [14, 15]. In addition, some studies showed biological variations and different spectrums of pathophysiology between early- and late-onset preeclampsia [18–20].

Clinical factors associated with risk of preeclampsia included primiparity, advanced maternal age, previous preeclampsia, family history of preeclampsia, multiple gestation, obesity, African-American race, diabetes mellitus, chronic hypertension, chronic renal disease, and presence of antiphospholipid antibodies were identified [21, 22]. Besides, there were evidences that stroke and hyperthyroidism increased the risk of preeclampsia [23, 24]. Further studies comparing predisposing factors of early- versus late-onset preeclampsia demonstrated similar risk factors but the strengths of association were differed among the factors. There were only two population-based studies, to our knowledge to evaluate each risk factor between early- and late-onset preeclampsia [14, 16]. One study, carried out by Lisonkova et al. revealed African-American race, chronic hypertension, and older age were more strongly associated with early-onset disease, whereas women with nulliparity and diabetes mellitus had higher risk to develop late-onset disease [14]. The other study conducted by Iacobelli et al. showed older age and higher prevalence of chronic hypertension in the group of early-onset disease [16].

The incidence of preeclampsia in Taiwan was significantly increased from 0.87 to 1.21% between 1998 and 2010 [25]. However, there was limited data of early- and late-onset preeclampsia rate and the associated factors in Taiwan has not been determined yet. The aim of the study was to investigate the population-based trends of early- and late-onset preeclampsia and examine the maternal risk factors in Taiwanese population.

## Methods

### Study design and data source

The population-based cohort study retrospectively included all ≧20 weeks singleton deliveries, comprising live births and stillbirths in Taiwan from January 1, 2001 to December 31, 2014. Two databases were used to obtain data electronically in this study. One was Taiwanese birth registration system in Health Promotion Administration, Ministry of Health and Welfare (https://www.hpa.gov.tw/EngPages/Index.aspx), which included information of birth date, gestational age, and fetal weights of all neonates and stillbirths with gestational age ≧20 weeks. Another was National Health Insurance Database (NHIRD, https://nhird.nhri.org.tw/en/index.html), which could be linked from Taiwanese birth registration system for details of maternal general data and diagnosis. Both databases have been encrypted to generate the unique identification to anonymous identification.

In Taiwan, birth certificates of all neonates and stillbirths with gestational age ≧20 weeks or gestational age < 20 weeks but fetal birth weights ≧500 g are issued at the hospital or medical institution and registered in the Ministry of Health and Welfare. The birth register can be linked to NHIRD, which was a database contained information of insured people, including demographic data, dates of hospitalization and clinical visits, and diagnostic codes as International Classification of Diseases, the 9th version (ICD-9) during the study period.

### Participants

There were 2,973,989 registered deliveries in Taiwan from January 1, 2001 to December 31, 2014. Women who were younger than 15-year-old or older than 55-year-old, with multiple gestation including twins, triplets, or quadruplets, and with gestational age at delivery less than 20 weeks were excluded. To identify the incidences of early- and late-onset preeclampsia, all the included delivers were categorized to ongoing pregnancy at 20 weeks of gestation and 34 weeks of gestation according to the gestational age at delivery, and each of which were the denominators of early- and late-onset preeclampsia rate, respectively [14, 15, 26].

**Fig. 1:**
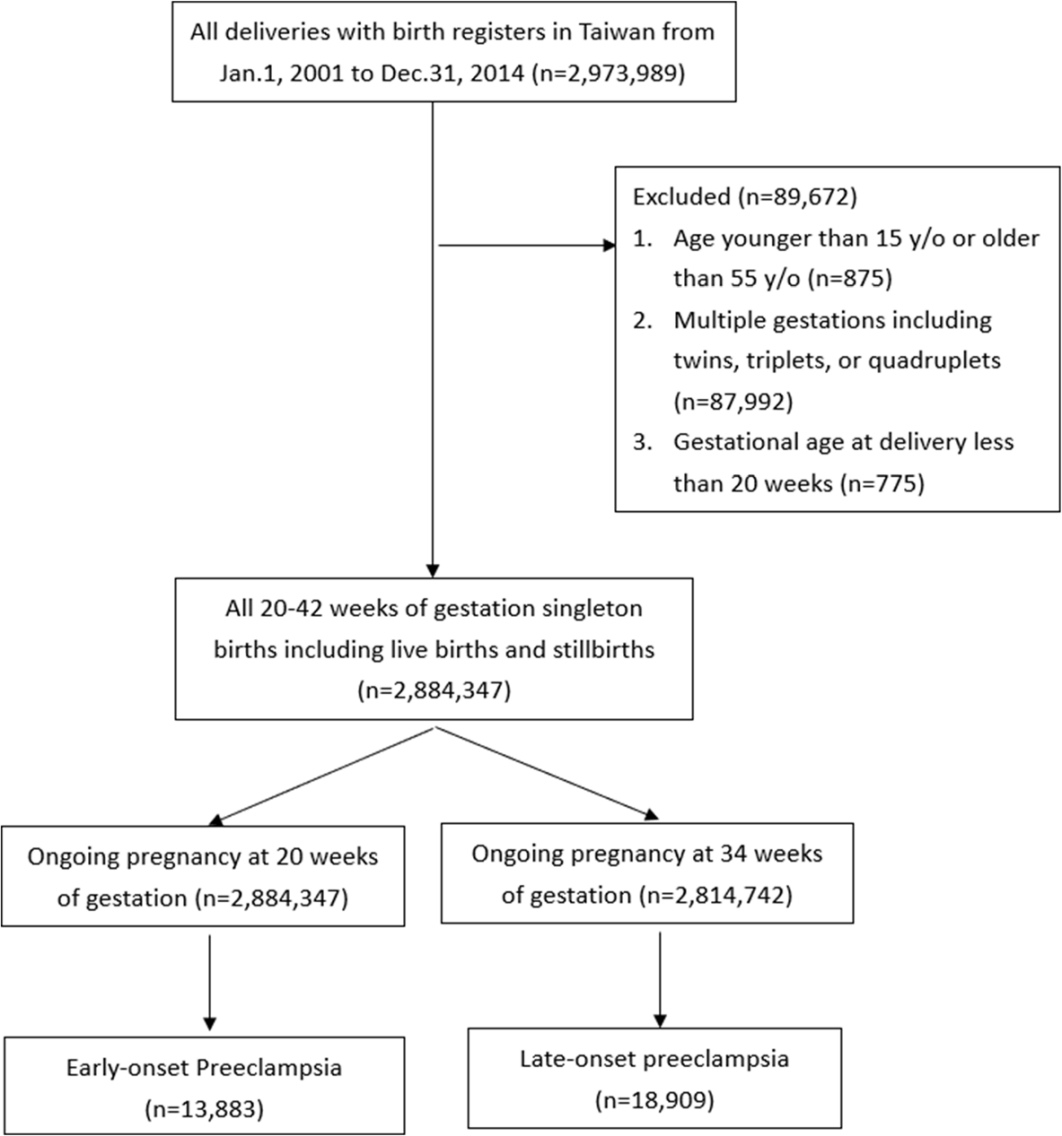
Flow chart of the study

The diagnostic criteria of preeclampsia was two occasions of hypertension at least 140/90 mmHg after 20 weeks of gestation accompanied by proteinuria > 300 mg/day or ≧1+ on dipstick based on International Society for the Study of Hypertension in Pregnancy (ISSHP) by 2014 [10, 26, 27]. All included delivers associated with diagnosis of preeclampsia were identified from NHIRD ICD-9 diagnostic codes 642.4, 6424.5, 6424.6, and 6424.7 [14, 15]. To obtain the gestational week of onset of preeclampsia for determing early- and late-onset preeclampsia, which were occurring less than and later than 34 weeks of gestation, the date of diagnosis was subtracted from the date at delivery, and calculated the gestational age according to the information of gestational weeks at birth in birth registers. A flow chart illustrating patient inclusion is showed in Fig. 1. The study was approved by Institutional Review Board of the Chang Gung Memorial Hospital (IRB No.201600657B0).

### Definition of variables

Maternal characteristics including maternal age (15–24, 25–34, and 35–55 years old), parity (number of prior**≧**20 weeks births, 0 vs. **≧1**), place of residence (urban, suburban, rural), income (quintiles 1 to 5), and clinical factors associated with risk of preeclampsia were examined for potential variables [14, 16, 22–24, 28, 29]. The clinical factors were identified in NHIRD, including acute coronary syndrome (ICD-9 codes, 410, 412 and 413), chronic ischemic heart disease (ICD-9 codes, 4140, 4148 and 4149), stroke (ICD-9 codes, 43,301, 43,311, 43,321, 43,331, 43,381, 43,391, 43,401, 43,411, 43,491, 435, 436, 4371, 4379 and 438), diabetes mellitus (ICD-9, 250), chronic hypertension (ICD-9 codes, 401–405) and hyperthyroidism (ICD-9 codes, 242).

### Statistical analysis

The standardized preeclampsia incidence was adjusted based on the age distribution in 2014 and 95% confidence intervals (CI) were derived from the Poisson distribution. The linear trends in proportion were assessed using Joinpoint Regression Program version 4.2.0.2 (National Cancer Institute, Bethesda, MD, USA) to estimate temporal trends in standardized incidence of preeclampsia. Bayesian information criterion was used to generate ‘joinpoints’ over time according to the changes of trend and average annual percentage change (AAPC) and 95%CI for each segment were calculated.

Multivariate logistic regression was used to obtain adjusted relative risks (ARR) and 95% CIs adjusted for the variables including maternal characteristics and clinical factors to examine the association with preeclampsia. The association between the clinical risk factors and early- or late-onset preeclampsia was further compared through the ratio of relative risks (RRR). Analyses were carried out using SAS, version 9.4 (SAS Institute, Cary, NC, USA). A 2-tailed *P* < 0.05 was considered significant. All tests of statistical hypothesis were done on the 2-sided 5% level of significance.

## Results

Among 2,973,989 delivers over the 14 years period, 2,884,347 delivers were included and 32,742 preeclampsia events were identified. The subgroups of early- and late- onset preeclampsia were 13,833 (42.3%) and 18,909 (57.6%) deliveries, respectively (Fig. 1).

The overall preeclampsia incidence increased from 1.1% (95%CI, 1.1–1.2) in 2001 to 1.7% (95%CI, 1.6–1.8) in 2014. Table 1 reveals crude and age-adjusted overall preeclampsia rates each year in the study period. The analysis of the overall, early-onset, and late-onset incidence trends through average annual percentage change is presented in Table 2. The overall preeclampsia rate was slightly increased from 1.1% (95%CI, 1.1–1.2) in 2001 to 1.3% (95%CI, 1.2–1.3) in 2012 with average annual percentage change (AAPC) 0.1%/year (95%CI, 0–0.2%). However, the incidence was remarkably increased from 1.3% (95%CI, 1.3–1.4) in 2012 to 1.7% (95%CI, 1.6–1.8) in 2014 with AAPC 1.3%/year (95%CI,0.3–2.5).

**Table 1:** Preeclampsia incidence between January 1, 2001 and December 31, 2014 (*n* = 2,884,347)

	Total						Early onset						Late onset					
Year	Total Delivery (n)	No of event	Crude IR, per 1000 births (95% CI)		Standardised IR, per 1000 births (95%CI)		Ongoing pregnancies at 20 weeks (n)	No of event	Crude IR, per 1000 births (95% CI)		Standardised IR, per 1000 births (95%CI)		Ongoing pregnancies at 34 weeks (n)	No of event	Crude IR, per 1000 births (95% CI)		Standardised IR, per 1000 births (95%CI)	
2001	243,357	2150	8.83	(8.46–9.21)	11.1	(10.5–11.6)	243,357	838	3.44	(3.21–3.68)	4.68	(4.31–5.05)	237,933	1312	5.51	(5.22–5.81)	6.56	(6.13–6.98)
2002	237,134	2186	9.22	(8.83–9.60)	11.6	(11.0–12.1)	237,134	950	4.01	(3.75–4.26)	5.37	(4.97–5.76)	231,862	1236	5.33	(5.03–5.63)	6.36	(5.93–6.78)
2003	221,232	2077	9.39	(8.98–9.79)	11.9	(11.3–12.5)	221,232	843	3.81	(3.55–4.07)	5.16	(4.76–5.56)	216,470	1234	5.70	(5.38–6.02)	6.88	(6.43–7.33)
2004	213,433	2014	9.44	(9.02–9.85)	11.7	(11.2–12.3)	213,433	839	3.93	(3.66–4.20)	5.10	(4.71–5.49)	208,461	1175	5.64	(5.31–5.96)	6.82	(6.37–7.27)
2005	203,137	1950	9.60	(9.17–10.0)	11.7	(11.1–12.3)	203,137	765	3.77	(3.50–4.03)	4.89	(4.51–5.27)	198,580	1185	5.97	(5.63–6.31)	6.98	(6.54–7.43)
2006	201,335	2306	11.5	(11.0–11.9)	13.3	(12.7–13.9)	201,335	968	4.81	(4.51–5.11)	5.76	(5.36–6.16)	196,617	1338	6.81	(6.44–7.17)	7.76	(7.30–8.23)
2007	199,389	2278	11.4	(11.0–11.9)	12.7	(12.1–13.3)	199,389	961	4.82	(4.51–5.12)	5.61	(5.22–5.99)	194,687	1317	6.76	(6.40–7.13)	7.27	(6.85–7.70)
2008	192,819	2086	10.8	(10.4–11.3)	11.9	(11.4–12.4)	192,819	891	4.62	(4.32–4.92)	5.22	(4.85–5.58)	188,125	1195	6.35	(5.99–6.71)	6.86	(6.45–7.28)
2009	188,735	2192	11.6	(11.1–12.1)	12.7	(12.2–13.3)	188,735	943	5.00	(4.68–5.32)	5.58	(5.21–5.95)	184,184	1249	6.78	(6.41–7.16)	7.32	(6.90–7.75)
2010	162,932	1927	11.8	(11.3–12.4)	12.4	(11.8–13.0)	162,932	801	4.92	(4.58–5.26)	5.23	(4.86–5.60)	158,724	1126	7.09	(6.68–7.51)	7.38	(6.94–7.82)
2011	194,095	2460	12.7	(12.2–13.2)	13.3	(12.7–13.8)	194,095	969	4.99	(4.68–5.31)	5.31	(4.97–5.65)	189,049	1491	7.89	(7.49–8.29)	8.18	(7.76–8.60)
2012	228,774	2885	12.6	(12.2–13.1)	13.0	(12.5–13.5)	228,774	1204	5.26	(4.97–5.56)	5.48	(5.17–5.79)	223,249	1681	7.53	(7.17–7.89)	7.73	(7.36–8.10)
2013	191,022	2775	14.5	(14.0–15.1)	14.6	(14.1–15.2)	191,022	1185	6.20	(5.85–6.56)	6.26	(5.91–6.62)	185,916	1590	8.55	(8.13–8.97)	8.59	(8.17–9.02)
2014	206,953	3506	16.9	(16.4–17.5)	16.9	(16.4–17.5)	206,953	1726	8.34	(7.95–8.73)	8.34	(7.95–8.73)	200,885	1780	8.86	(8.45–9.27)	8.86	(8.45–9.27)

*Abbreviation*: *IR* incidence rate

**Table 2:** Joinpoint analysis of trend of preeclampsia incidence in Taiwan between 2001 and 2014

	Standardised IR, per 1000 births		AAPC		Trend 1			Trend 2		
	2001	2014			Year	AAPC (95%CI)		Year	AAPC (95%CI)	
Trend of entire cohort	11.1 (10.5–11.6)	16.9 (16.4–17.5)	3.01	(1.50 to 4.54)*	2001–2012	1.25	(0.36 to 2.14)*	2012–2014	13.3	(2.55 to 25.1) *
by onset weeks										
< 34 weeks	4.68 (4.31–5.05)	8.34 (7.95–8.73)	3.67	(1.67 to 5.72)*	2001–2012	0.58	(− 0.63 to 1.80)	2012–2014	22.5	(7.50 to 39.6) *
≥ 34 weeks	6.56 (6.13–6.98)	8.86 (8.45–9.27)	2.21	(1.51 to 2.91)*						

*AAPC* average annual percent change

*P < 0.05

**Fig. 2:**
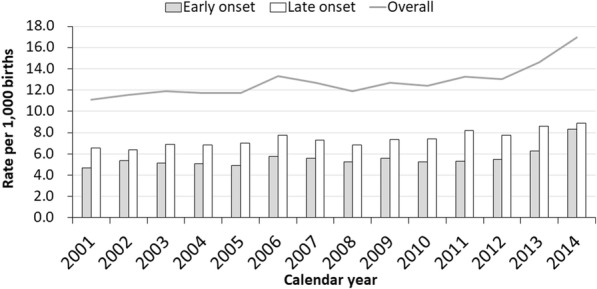
Trends in overall, early-onset, and late-onset preeclampsia between 2001 and 2014

Over the 14 years study period, the incidence trends in late-onset preeclampsia was steadily increasing from 0.7% (95%CI, 0.6–0.7) in 2001 to 0.9% (95%CI, 0.8–0.9) in 2014 with AAPC 0.2%/year (95%CI, 0.2–0.3). However, in early-onset preeclampsia, similar to the trend of overall preeclampsia, the incidence was relatively steady around 0.5% (95%CI, 0.4–0.5) in 2001 and 0.5% (95%CI, 0.5–0.6) in 2012 but predominantly increase from 0.5% (95%CI, 0.4–0.5) in 2012 to 0.8% (95%CI, 0.8–0.9) in 2014 with AAPC 2.3%/year (95%CI, 0.8–4.0). Figure 2 illustrates the trends in overall preeclampsia, early-onset, and late-onset disease.

Maternal characteristics (age, parity, place of residence, and income) and clinical factors (acute coronary syndrome, chronic ischemic heart disease, stroke, diabetes mellitus, chronic hypertension, and hyperthyroidism) are described in Table 3. In multivariate logistic regression, all maternal characteristics and clinical factors listed in Table 3 were adjusted as possible confounders. Women with older age, nulliparity, chronic hypertension, diabetes mellitus, stroke, and hyperthyroidism were more likely to develop preeclampsia (all *p* values< 0.01), while acute coronary syndrome and chronic ischemic heart disease were not significantly associated with preeclampsia. The ARR of each clinical factor of overall preeclampsia are displayed in Table 4. Of note, women with chronic hypertension had much higher risk of preeclampsia (ARR, 12.1; 95%CI, 11.5–12.8).

**Table 3:** Maternal characteristics and clinical factors associated with early- and late-onset preeclampsia

Characteristics	Ongoing pregnancies at 20 weeks	Early-onset preeclampsia			Ongoing pregnancies at 34 weeks	Late-onset preeclampsia		
	*n* = 2,884,347	*n* = 13,883	Rate per 1000 (95% CI)		*n* = 2,814,742	*n* = 18,909	Rate per 1000 (95% CI)	
Age at delivery								
15–24	525,457	1305	2.48	(2.35–2.62)	513,581	2447	4.76	(4.58–4.95)
25–34	1,956,147	8688	4.44	(4.35–4.53)	1,914,210	12,155	6.35	(6.24–6.46)
35–55	402,743	3890	9.66	(9.36–9.96)	386,951	4307	11.13	(10.8–11.5)
Number of prior ≧20 weeks births (parity)								
0	1,938,341	9559	4.93	(4.83–5.03)	1,893,197	14,611	7.72	(7.59–7.84)
≥ 1	946,006	4324	4.57	(4.43–4.71)	921,545	4298	4.66	(4.52–4.80)
Place of residence								
Urban	1681,534	7988	4.75	(4.65–4.85)	1,641,570	11,169	6.8	(6.68–6.93)
Suburban	826,656	4368	5.28	(5.13–5.44)	806,062	5645	7	(6.82–7.19)
Rural	192,141	1027	5.35	(5.02–5.67)	186,732	1403	7.51	(7.12–7.91)
Unknown	184,016	500	2.72	(2.48–2.96)	180,378	692	3.84	(3.55–4.12)
Income levels								
Quintile 1	558,314	2689	4.82	(4.63–5.00)	542,450	3825	7.05	(6.83–7.27)
Quintile 2	516,131	2560	4.96	(4.77–5.15)	503,177	3441	6.84	(6.61–7.07)
Quintile 3	566,360	3097	5.47	(5.28–5.66)	553,016	4140	7.49	(7.26–7.71)
Quintile 4	527,626	2508	4.75	(4.57–4.94)	516,050	3499	6.78	(6.56–7.01)
Quintile 5	540,007	2554	4.73	(4.55–4.91)	527,737	3351	6.35	(6.13–6.56)
Unknown	175,909	475	2.7	(2.46–2.94)	172,312	653	3.79	(3.50–4.08)
Acute Coronary syndrome								
No	2,878,429	13,809	4.8	(4.72–4.88)	2,809,040	18,854	6.71	(6.62–6.81)
Yes	5918	74	12.5	(9.66–15.4)	5702	55	9.65	(7.10–12.2)
Chronic ischemic heart disease								
No	2,877,398	13,767	4.78	(4.70–4.86)	2,808,117	18,801	6.7	(6.60–6.79)
Yes	6949	116	16.69	(13.7–19.7)	6625	108	16.3	(13.2–19.4)
Stroke								
No	2,877,911	13,792	4.79	(4.71–4.87)	2,808,569	18,824	6.7	(6.61–6.80)
Yes	6436	91	14.14	(11.2–17.0)	6173	85	13.77	(10.8–16.7)
Diabetes mellitus								
No	2,856,198	13,164	4.61	(4.53–4.69)	2,788,463	18,256	6.55	(6.45–6.64)
Yes	28,149	719	25.54	(23.7–27.4)	26,279	653	24.85	(22.9–26.8)
Chronic hypertension								
No	2,862,222	11,671	4.08	(4.00–4.15)	2,796,074	17,471	6.25	(6.16–6.34)
Yes	22,125	2212	99.98	(95.8–104)	18,668	1438	77.03	(73.0–81.0)
Hyperthyroidism								
No	2,813,030	13,316	4.73	(4.65–4.81)	2,745,618	18,231	6.64	(6.54–6.74)
Yes	71,317	567	7.95	(7.30–8.60)	69,124	678	9.81	(9.07–10.5)

**Table 4:** Maternal characteristics and clinical risk factors associated with preeclampsia

	Incidence rate per 1000 births (95%CI)		Crude Relative risk (95%CI)		*P* Value	Adjusted Relative risk (95%CI)^a^		*P* Value
Age at delivery								
15–24	7.19	(6.96–7.42)	Reference			Reference		
25–34	10.77	(10.6–10.9)	1.48	(1.43–1.54)	< 0.01*	1.49	(1.44-1.55)	< 0.01*
35–55	20.78	(20.3–21.2)	2.78	(2.67–2.89)	< 0.01*	2.62	(2.51-2.73)	< 0.01*
Number of prior ≧20 weeks births (parity)								
0	12.63	(12.5–12.8)	1.35	(1.32–1.38)	< 0.01*	1.71	(1.67-1.76)	< 0.01*
≥ 1	9.2	(9.00-9.39)	Reference			Reference		
Acute Coronary syndrome^b^								
No	11.48	(11.4–11.6)	Reference			Reference		
Yes	22.28	(18.4–26.1)	1.93	(1.60–2.31)	< 0.01*	0.9	(0.74-1.10)	0.32
Chronic ischemic heart disease								
No	11.45	(11.3–11.6)	Reference			Reference		
Yes	33.31	(28.9–37.7)	2.83	(2.46–3.27)	< 0.01*	0.9	(0.77-1.06)	0.21
Stroke								
No	11.46	(11.3–11.6)	Reference			Reference		
Yes	28.12	(24.0–32.3)	2.44	(2.09–2.85)	< 0.01*	1.33	(1.13-1.58)	< 0.01*
Diabetes mellitus								
No	11.12	(11.0–11.2)	Reference			Reference		
Yes	51.24	(48.5–53.9)	4.28	(4.02–4.55)	< 0.01*	2.01	(1.86-2.16)	< 0.01*
Chronic hypertension								
No	10.29	(10.2–10.4)	Reference			Reference		
Yes	197.56	(191–204)	15.5	(14.8–16.2)	< 0.01*	12.14	(11.5-12.8)	< 0.01*
Hyperthyroidism								
No	11.34	(11.2–11.5)	Reference			Reference		
Yes	17.77	(16.8–18.8)	1.56	(1.46–1.65)	< 0.01*	1.21	(1.14-1.29)	< 0.01*

^a^Adjusted Relative risk: adjusted with age at delivery, income, urbanization, parity, acute coronary syndrome, chronic ischemic heart disease, stroke, diabetes mellitus, chronic hypertension and hyperthyroidism

^b^Acute Coronary syndrome included myocardial infarction and unstable angina

*P < 0.05

For subgroup analysis of early- and late-onset preeclampsia, the clinical factors associated with early- or late-onset diseases were identical to overall preeclampsia, detailed in Table 5. To compare the strength of the association among the risk factors between early- and late-onset preeclampsia, RRR of each clinical factor was calculated according to the ARR of early- and late preeclampsia. Advanced maternal age (> 35 years) (RRR, 1.4; 95%CI, 1.3–1.5, *p* < 0.01) and chronic hypertension (RRR, 1.7; 95%CI, 1.6–1.9, p < 0.01) had higher risk to develop early-onset preeclampsia. In the contrary, primiparity (RRR, 0.7; 95%CI, 0.7–0.8, p < 0.01) was more strongly associated with late-onset disease. Other risk factors of preeclampsia including diabetes mellitus, stroke, and hyperthyroidism revealed no statistical difference in the association of early- and late-onset preeclampsia (Table 5).

**Table 5:** Maternal characteristics and clinical risk factors associated with early- and late-onset preeclampsia

	Early onset preeclampsia						Late onset preeclampsia						Ratio of Relative risk (95%CI)		*P* Value
	Crude Relative risk (95%CI)		*P* Value	Adjusted Relative risk (95%CI)^a^		*P* Value	Crude Relative risk (95%CI)		*P* Value	Adjusted Relative risk (95%CI)^a^		*P* Value			
Age at delivery															
15–24	Reference			Reference			Reference			Reference			Reference		
25–34	1.79	(1.69–1.90)	< 0.01*	1.73	(1.63-1.84)	< 0.01*	1.33	(1.27-1.39)	< 0.01*	1.36	(1.30-1.43)	< 0.01*	1.27	(1.78-1.37)	< 0.01*
35–55	3.84	(3.60–4.10)	< 0.01*	3.21	(2.99-3.44)	< 0.01*	2.31	(2.19-2.42)	< 0.01*	2.28	(2.16-2.40)	< 0.01*	1.41	(1.29-1.54)	< 0.01*
Number of prior ≧20 weeks births (parity)															
0	1.06	(1.02–1.09)	< 0.01*	1.39	(1.34-1.44)	< 0.01*	1.65	(1.59-1.70)	< 0.01*	1.95	(1.89-2.02)	< 0.01*	0.71	(0.68-0.75)	< 0.01*
≥ 1	Reference			Reference			Reference			Reference			Reference		
Acute Coronary syndrome^b^															
No	Reference			Reference			Reference			Reference			Reference		
Yes	2.59	(2.04–3.28)	< 0.01*	1	(0.78-1.30)	0.98	1.45	(1.11–1.90)	< 0.01*	0.77	(0.58-1.02)	0.07	1.3	(0.89–1.90)	0.09
Chronic ischemic heart disease															
No	Reference			Reference			Reference			Reference			Reference		
Yes	3.44	(2.83–4.17)	< 0.01*	0.84	(0.68-1.04)	0.11	2.45	(2.02–2.98)	< 0.01*	0.92	(0.74-1.14)	0.45	0.91	(0.67–1.24)	0.28
Stroke															
No	Reference			Reference			Reference			Reference			Reference		
Yes	2.96	(2.37–3.68)	< 0.01*	1.33	(1.05-1.68)	0.02*	2.07	(1.67-2.58)	< 0.01*	1.28	(1.02-1.61)	0.03*	1.04	(0.75-1.44)	0.4
Diabetes mellitus															
No	Reference			Reference			Reference			Reference			Reference		
Yes	5.34	(4.91–5.81)	< 0.01*	1.9	(1.71-2.10)	< 0.01*	3.76	(3.46-4.08)	< 0.01*	2.03	(1.85-2.23)	< 0.01*	0.94	(0.82-1.08)	0.19
Chronic hypertension															
No	Reference			Reference			Reference			Reference			Reference		
Yes	22.73	(21.5–24.0)	< 0.01*	16.8	(15.7-18.0)	< 0.01*	12.21	(11.5-13.0)	< 0.01*	9.85	(9.19-10.6)	< 0.01*	1.71	(1.55-1.88)	< 0.01*
Hyperthyroidism															
No	Reference			Reference			Reference			Reference			Reference		
Yes	1.68	(1.53–1.83)	< 0.01*	1.18	(1.07-1.29)	< 0.01*	1.48	(1.36-1.60)	< 0.01*	1.21	(1.12-1.32)	< 0.01*	0.98	(0.86-1.10)	0.34

^a^Adjusted Relative risk: adjusted for with age at delivery, income, urbanization, parity, acute coronary syndrome, chronic ischemic heart disease, stroke, diabetes mellitus, chronic hypertension and hyperthyroidism

^b^Acute Coronary syndrome included myocardial infarction and unstable angina

*P < 0.05

## Discussion

The incidences of overall, and early- and late-onset preeclampsia were increasing in Taiwan between 2001 and 2014, predominantly for early-onset disease. Pregnant women with advanced maternal age, primiparity, chronic hypertension, stroke, diabetes mellitus, and hyperthyroidism had significantly higher risk of developing preeclampsia. Among the factors, older age and hypertension were more strongly associated with early-onset disease.

Our study has certain limitations. First, overall preeclampsia was identified by diagnostic codes; thus, the artificial coding error and misclassification bias could not be avoided. Second, NHIRD included 99% of Taiwanese residents, which led to around 1.2% deliveries in birth registration not be linked to hospital records. The missing data could be susceptible to underascertainment, resulting in slightly underestimation of the incidence. Third, if a patient had neither antenatal exam nor delivered in hospitals, or developed postpartum preeclampsia without hospitalization, the diagnostic code could not be obtained from the hospital record. The such undetermined were few and less severe but may cause underestimation as well. Fourth, we did not classify the severity of preeclampsia or determine the subgroup using aspirin for prevention, both of which may give more information to interpretate the trends of preeclampsia. In addition, we failed to assess certain risk factors including BMI, preeclampsia history in prior pregnancy, and family history of preeclampsia, as well as the maternal and neonatal outcomes due to limited information.

The overall preeclampsia rate in Taiwan was relatively lower than the worldwide studies though, 42.3% preeclampsia events identified as early-onset disease was remarkable. The incidence of preeclampsia was 1.1 to 1.7% in Taiwan compared with 2 to 8% incidence of preeclampsia worldwide with regional variations [2]. The lower incidence could be owing to the majority of Asian race in the population, which had lower risk of developing preeclampsia, 2.0–3.0%according to previous studies [26, 30]. We did not collect data of races in this study but over 95% of the population is Han Chinese, who are regarded as East Asian ethnic group based on the data in Ministry of Interior. Besides, there could possibly be slight underestimation because of missing data in NHIRD or the artificial bias of disease coding. Nevertheless, NHIRD was one of the powerful tools in assessment of the epidemiology in Taiwan because of the high coverage of National Health Insurance program, financing around 99% Taiwanese residents [31]. In the current study, the unidentified anonymous identification between birth register system and NHIRD was around 1.2%. In addition, compared with the study by Chan et al., who revealed the increased incidence of preeclampsia in Taiwan was notable from 0.87 to 1.21% between 1998 and 2010 [25]. Therefore, the interpretation of exact preeclampsia rate could be somewhat underestimated owing to the unavoidable bias but the persisted increasing incidence of preeclampsia in the population-based study was thoroughly informative.

Our data suggested that the trend of preeclampsia was increasing between 2001 and 2014 after age ajustment, especially in early-onset disease from 2012 to 2014. We analyzed the subgroup trends of preeclampsia depend on maternal age and women with or without hypertension, respectively [Additional file 1 and Additional file 2]. Additional file 1 shows increased trend in all three age groups (age 15–25, 25–35, and 35–55). However, Additional file 2 reveals no significant incidence change in women with our without hypertension during the study period. The subgroup analysis indicated that the increasing trend of preeclampsia occurred in all age groups and possibly the growing number of women with hypertension. Interestingly, the rise rate of preeclampsia was not universally consistent, for instance, studies for the entire USA from 1999 to 2004 showed plateaued rate [32] and in Western New York from 1999 to 2003 [33] and European countries during the past ten years [34] reported slight declines in preeclampsia. However, the increase in preeclampsia in our population could partially affected by the revision of diagnostic criteria. American College of Obstetrician and Gynecologist (ACOG) in2013 and ISSHP in 2014 have excluded proteinuria as an necessary condition to establish diagnosis of preeclampsia in women presence of organ dysfunction of uteroplacental dysfunction [35, 36]. Therefore, the observation of significant rise in early-onset preeclampsia from 2012 to 2014 in this study was a conservative indication of increasing trend but the true percentage change should be followed since overall revision of the criteria in Taiwan.

The proportion of early-onset preeclampsia was significantly higher (42.3% in early-onset and 57.6% in late onset disease) than previous studies conducted other than Taiwan [14–16], about twofold to nine-fold of late-onset disease than early-onset preeclampsia. The difference could attributed to increasing prevalence of chronic hypertension as a result of a surge of risk rates of prehypertension, obesity and metabolic syndrome in Taiwan [37]. Genetic variation or epigenetic regulation such as DNA methylation or microRNA expression associated with preeclampsia in the population [18, 38–40], and the theory of developmental origins of health and disease (DOHaD) that the early life environment impacting the risk of chronic disease from childhood to adulthood [41] could possibly cause the population vulnerable to early- or late-onset preeclampsia. However, none of the hypotheses has been verified. Therefore, higher proportion, almost half of early-onset preeclampsia women in the population warranted further investigation to provide addition insights into the variation of early- and late-onset preeclampsia incidences.

In the population between 2001 and 2014, our findings of the risk factors including advanced maternal age, primiparity, stroke, diabetes mellitus, chronic hypertension, or hyperthyroidism were consistent with commonly quoted clinical factors of preeclampsia [14, 16, 21, 22]. The subgroups of women with early- and late-onset preeclampsia were similar in terms of maternal risk factors but different association in age, parity, and chronic hypertension. Among the risk factors, advanced maternal age and chronic hypertension revealed stronger association with early-onset disease, while primiparity had higher risk of late-onset preeclampsia. The findings were similar to previous studies [14, 16]. To assess the possible interaction of old age and hypertension. We divided women according to different age groups (age 15–25. age 25–35, age 35–55)and women with or without hypertension, respectively [Additional file 3]. In Additional file 3: Table S6 reveals that chronic hypertension had highest ARR in all age groups and demonstrates old age was an independent risk factor either in women with or without hypertension. The different strength of association among the specific risk factors between early- and late-onset preeclampsia could be associated with the different pathophysiologic mechanisms including histology, hemodynamic change, or vascular adaption between early- and late-onset preeclampsia. Several evidences supported more typical histological change of defective trophoblast invasion and higher percentage of altered uterine artery Doppler in early-onset disease [18–20]. Cheng et al. revealed maternal serum markers associated with cardiovascular inflammatory response such as high-sensitive C-reactive protein and homocysteine were significantly higher in early-onset preeclampsia, which could be related to direct injury to vascular endothelial cells or increased oxidative stress and resulting in the sequence of placenta dysfunction and poorer outcomes [42]. Despite those evidences, there was yet no definite pathophysiology and mechanism to explain the development towards early- or late-onset preeclampsia. Thus, more investigations are needed to identify the specific correlation between old age or chronic hypertension and early-onset preeclampsia, and clinicians should be aware of preeclampsia prediction in women with clinical risk factors, particularly chronic hypertension, which had highest risk (ARR, 16.8, 95%CI, 15.7–18.0) of early-onset preeclampsia in the current study. Furthermore, early intervention such as aspirin prophylaxis may be considered in patients with evidence of higher risk, according to the ASPRE trial [43] to prevent severe maternal morbidities and poorer birth outcomes of early-onset preeclampsia [14, 16, 17]. On the other hand, Valensise et al. have found late-onset preeclampsia appeared to be more frequent in patients with high body mass index (BMI) compared with early-onset disease [19]. However, we had no data of maternal BMI to assess and the mechanism between preeclampsia and obesity was yet completely understood. In general, maternal risk factors between early- and late- onset preeclampsia were similar but old age and chronic hypertension appeared stronger association to early-onset disease and primiparity had higher risk of late-onset preeclampsia.

This study was the first documented the increasing trend of preeclampsia in Taiwan between 2001 and 2014, predominantly in early-onset disease. The strength of the study includes a large cohort sample from a specific geographic area which is very representative of the regional population and the large study size provided reliability in statistics.

## Conclusions

In conclusion, the population-base study showed a rise in the incidence of preeclampsia, particularly in early-onset disease in Taiwan from 2001 to 2014, suggesting the clinical early prediction, identification, and management of the diseases will increasingly challenge obstetricians. As increasing number of advanced maternal age and chronic hypertension in delivering population in Taiwan, preconceptional counseling and surveillance is warranted in pregnant women with higher risk of early-onset preeclampsia. Further study of the predominance of early-onset preeclampsia in the population and the stronger association with old age and hypertension and early-onset preeclampsia is required.

## Additional files

Additional file 1 Trends of preeclampsia incidence according to different ages between 2001 and 2014 (blue: 15–25-year-old; red: 25–35-year-old; green:35–55-year-old). (TIF 1329 kb)

Additional file 2 Trends of preeclampsia incidence according to women with or without hypertension (orange: women with hypertension; blue: women without hypertension). (TIF 1131 kb)

Additional file 3 **Table S6** Maternal characteristics and clinical risk factors associated with preeclampsia by age group. **Table S7** Maternal characteristics and clinical risk factors associated with preeclampsia by hypertension. (DOCX 22 kb)

## Abbreviations

AAPC: average annual percentage change
ACOG: American College of Obstetrician and Gynecologist
ARR: adjusted relative risk
BMI: body mass index
CI: confidence interval
DOHaD: developmental origins of health and disease
ICD-9: International Classification of Diseases, the 9th version
ISSHP: International Society for the Study of Hypertension in Pregnancy
NHIRD: National Health Insurance Database
RRR: ratio of relative risk
WHO: World Health Organization
