# Supplementary figures and images for: Population-based trends and risk factors of early- and late-onset preeclampsia in Taiwan 2001–2014
Source: BMC Pregnancy Childbirth. 2018 May 31;18:199. doi: 10.1186/s12884-018-1845-7 (PMC5984409; doi:10.1186/s12884-018-1845-7)

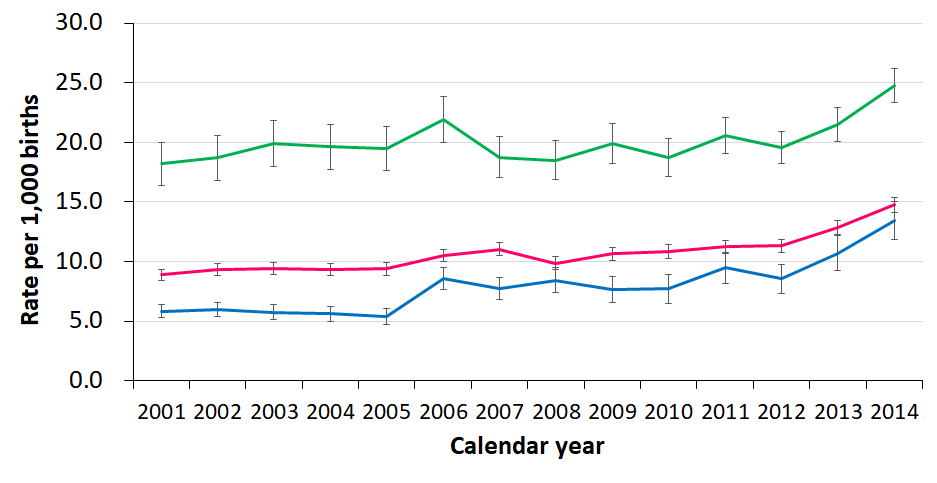

Supplement: Supplementary file 1 — Trends of preeclampsia incidence according to different ages between 2001 and 2014 (blue: 15–25-year-old; red: 25–35-year-old; green:35–55-year-old). (TIF 1329 kb) [file 12884_2018_1845_MOESM1_ESM.tif]

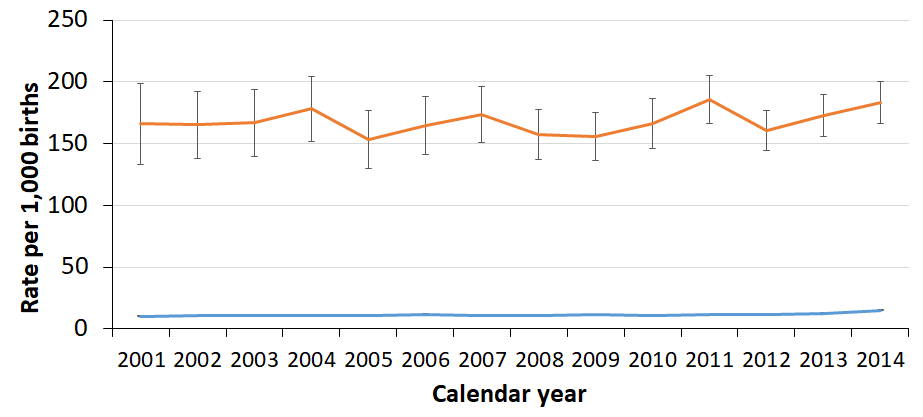

Supplement: Supplementary file 2 — Trends of preeclampsia incidence according to women with or without hypertension (orange: women with hypertension; blue: women without hypertension). (TIF 1131 kb) [file 12884_2018_1845_MOESM2_ESM.tif]
