# Supplementary material for: Population-based trends and risk factors of early- and late-onset preeclampsia in Taiwan 2001–2014
Source: BMC Pregnancy Childbirth. 2018 May 31;18:199. doi: 10.1186/s12884-018-1845-7 (PMC5984409; doi:10.1186/s12884-018-1845-7)
Supplement: Supplementary file 3 — Table S6 Maternal characteristics and clinical risk factors associated with preeclampsia by age group. Table S7 Maternal characteristics and clinical risk factors associated with preeclampsia by hypertension. (DOCX 22 kb) [file 12884_2018_1845_MOESM3_ESM.docx]

**Table 6. Maternal characteristics and clinical risk factors associated with preeclampsia by age group**

|  | **Age 15-24** | | |  | **Age 25-34** | | |  | | **Age 35-55** | | |
| --- | --- | --- | --- | --- | --- | --- | --- | --- | --- | --- | --- | --- |
|  | **Adjusted Relative risk**  **(95%CI)‡** | | ***P* value** |  | **Adjusted Relative risk**  **(95%CI)‡** | | ***P* value** |  | | **Adjusted Relative risk**  **(95%CI)‡** | | ***P* value** |
| **Number of prior ≧20 weeks births (parity)** |  |  |  |  |  |  |  |  | |  |  |  |
| 0 | 1.88 | (1.71-2.06) | <0.01* |  | 1.69 | (1.64-1.75) | <0.01* |  | | 1.63 | (1.56-1.71) | <0.01* |
| ≥1 | Reference | |  |  | Reference | |  |  | | Reference | |  |
| **Acute Coronary syndrome** |  |  |  |  |  |  |  |  | |  |  |  |
| No | Reference | |  |  | Reference | |  |  | | Reference | |  |
| Yes | 0.81 | (0.33-1.99) | 0.64 |  | 0.89 | (0.67-1.18) | 0.41 |  | | 0.95 | (0.71-1.26) | 0.72 |
| **Chronic ischemic heart disease** |  |  |  |  |  |  |  |  | |  |  |  |
| No | Reference | |  |  | Reference | |  |  | | Reference | |  |
| Yes | 0.70 | (0.32-1.53) | 0.37 |  | 0.83 | (0.66-1.04) | 0.10 |  | | 0.97 | (0.77-1.23) | 0.80 |
| **Previous stroke** |  |  |  |  |  |  |  |  | |  |  |  |
| No | Reference | |  |  | Reference | |  |  | | Reference | |  |
| Yes | 1.06 | (0.47-2.37) | 0.89 |  | 1.31 | (1.04-1.64) | 0.02* |  | | 1.39 | (1.08-1.78) | 0.01* |
| **Diabetes mellitus** |  |  |  |  |  |  |  |  | |  |  |  |
| No | Reference | |  |  | Reference | |  |  | | Reference | |  |
| Yes | 4.79 | (3.56-6.46) | <0.01* |  | 2.17 | (1.97-2.39) | <0.01* |  | | 1.64 | (1.47-1.84) | <0.01* |
| **Chronic hypertension** |  |  |  |  |  |  |  |  | |  |  |  |
| No | Reference | |  |  | Reference | |  |  | | Reference | |  |
| Yes | 10.24 | (7.64-13.7) | <0.01* |  | 14.83 | (13.9-15.8) | <0.01* |  | | 11.19 | (10.4-12.0) | <0.01* |
| **Hyperthyroidism** |  |  |  |  |  |  |  |  |  | |  |  |
| No | Reference | |  |  | Reference | |  |  | Reference | | |  |
| Yes | 1.92 | (1.53-2.42) | <0.01* |  | 1.24 | (1.15-1.34) | <0.01* |  | 1.04 | | (0.93-1.17) | 0.46 |

‡Adjusted Relative risk: adjusted with income, urbanization, parity, acute coronary syndrome, chronic ischemic heart disease, stroke, diabetes mellitus, chronic hypertension and hyperthyroidism

**P*<0.05

**Table 7.** **Maternal characteristics and clinical risk factors associated with preeclampsia by hypertension**

|  | **Without HTN** | | | | | | |  | | **With HTN** | | | | | | |
| --- | --- | --- | --- | --- | --- | --- | --- | --- | --- | --- | --- | --- | --- | --- | --- | --- |
|  | **Crude Relative risk**  **(95%CI)** | | **P value** |  | **Adjusted Relative risk**  **(95%CI)‡** | | ***P* value** |  | | **Crude Relative risk**  **(95%CI)** | | ***P* value** |  | **Adjusted Relative risk**  **(95%CI)‡** | | **P value** |
| **Age at deliver** |  |  |  |  |  |  |  |  | |  |  |  |  |  |  |  |
| 15-24 | Reference | |  |  | Reference | |  |  | | Reference | |  |  | Reference | |  |
| 25-34 | 1.40 | (1.35-1.45) | <0.01* |  | 1.47 | (1.41-1.53) | <0.01* |  | | 1.57 | (1.30-1.90) | <0.01* |  | 1.61 | (1.32-1.95) | <0.01* |
| 35-55 | 2.46 | (2.36-2.56) | <0.01* |  | 2.68 | (2.56-2.80) | <0.01* |  | | 1.84 | (1.51-2.24) | <0.01* |  | 1.90 | (1.56-2.32) | <0.01* |
| **Number of prior ≧20 weeks births (parity)** |  |  |  |  |  |  |  |  | |  |  |  |  |  |  |  |
| 0 | 1.53 | (1.50-1.57) | <0.01* |  | 1.75 | (1.70-1.80) | <0.01* |  | | 1.02 | (0.96-1.09) | 0.51 |  | 1.04 | (0.97-1.11) | 0.28 |
| ≥1 | Reference | |  |  | Reference | |  |  | | Reference | |  |  | Reference | |  |
| **Acute Coronary syndrome** |  |  |  |  |  |  |  |  | |  |  |  |  |  |  |  |
| No | Reference | |  |  | Reference | |  |  | | Reference | |  |  | Reference | |  |
| Yes | 1.25 | (0.99-1.60) | 0.06 |  | 1.07 | (0.84-1.36) | 0.60 |  | | 0.73 | (0.55-0.97) | 0.03* |  | 0.73 | (0.55-0.97) | 0.03* |
| **Chronic ischemic heart disease** |  |  |  |  |  |  |  |  | |  |  |  |  |  |  |  |
| No | Reference | |  |  | Reference | |  |  | | Reference | |  |  | Reference | |  |
| Yes | 1.67 | (1.36-2.05) | <0.01* |  | 1.36 | (1.11-1.67) | <0.01* |  | | 0.70 | (0.57-0.85) | <0.01* |  | 0.70 | (0.57-0.85) | <0.01* |
| **Previous stroke** |  |  |  |  |  |  |  |  | |  |  |  |  |  |  |  |
| No | Reference | |  |  | Reference | |  |  | | Reference | |  |  | Reference | |  |
| Yes | 1.72 | (1.41-2.09) | <0.01* |  | 1.48 | (1.22-1.81) | <0.01* |  | | 1.07 | (0.82-1.39) | 0.62 |  | 1.10 | (0.84-1.43) | 0.48 |
| **Diabetes mellitus** |  |  |  |  |  |  |  |  | |  |  |  |  |  |  |  |
| No | Reference | |  |  | Reference | |  |  | | Reference | |  |  | Reference | |  |
| Yes | 3.21 | (2.98-3.47) | <0.01* |  | 2.93 | (2.72-3.17) | <0.01* |  | | 1.09 | (0.99-1.21) | 0.09 |  | 1.09 | (0.99-1.21) | 0.09 |
| **Hyperthyroidism** |  |  |  |  |  |  |  |  |  | |  |  |  |  |  |  |
| No | Reference | |  |  | Reference | |  |  | Reference | | |  |  | Reference | |  |
| Yes | 1.48 | (1.39-1.58) | <0.01* |  | 1.33 | (1.25-1.42) | <0.01* |  | 0.66 | | (0.56-0.77) | <0.01* |  | 0.65 | (0.56-0.76) | <0.01* |

‡Adjusted Relative risk: adjusted with age at delivery, income, urbanization, parity, acute coronary syndrome, chronic ischemic heart disease, stroke, diabetes mellitus, and hyperthyroidism

**P*<0.05
